# A lymphostatin homologue from *Chlamydia pecorum* inhibits mitogen-activated bovine T cell proliferation and IFNγ production

**DOI:** 10.1080/21505594.2025.2506500

**Published:** 2025-05-22

**Authors:** Elizabeth A. Blackburn, Cosmin Chintoan-Uta, Andrew G. Bease, Maarten W. Tuijtel, Max Hateley, Martin Wear, David Longbottom, Robin Cassady-Cain, Mark P. Stevens

**Affiliations:** a The Edinburgh Protein Production Facility, University of Edinburgh, Edinburgh, UK; b The Roslin Institute and Royal (Dick) School of Veterinary Studies, University of Edinburgh, Edinburgh, UK; c The Moredun Research Institute, Pentland Science Park, Edinburgh, UK

**Keywords:** *Escherichia coli*, lymphostatin, *Chlamydia*, inhibition, lymphocyte, IFNγ

## Abstract

Pathogens frequently produce proteins to evade or inhibit host immune responses. One such protein is lymphostatin from attaching and effacing *Escherichia coli* (also known as lymphocyte inhibitory factor A; LifA), which influences intestinal colonization and inhibits mitogen- and antigen-activated proliferation of T lymphocytes and pro-inflammatory cytokine synthesis. Here, we report the cloning, purification and characterization of a LifA homologue from *Chlamydia pecorum*. The predicted 382 KDa protein (CPE2_0552) exhibited 36% identity and 55% similarity over 3171 amino acids to lymphostatin from enteropathogenic *E. coli* strain E2348/69. CPE2_0552 shares glycosyltransferase and cysteine protease motifs required for lymphostatin activity, including similarity in the tertiary structure of these domains predicted by AlphaFold 3. Purified CPE2_0552 did not share the L-shaped globular structure of lymphostatin when analyzed by transmission electron microscopy. CPE2_0552 inhibited concanavalin A-stimulated proliferation of bovine T cells in a concentration-dependent manner, with an inhibitory dose 50 (ID_50_) of 812 pg/mL. This was 38-fold higher than the ID_50_ of *E. coli* E2348/69 lymphostatin tested in parallel on T cells from the same donors (21 pg/mL), but was similar to another LifA homologue from *E. coli* O157:H7 (ToxB). Moreover, CPE2_0552 inhibited the secretion of interferon gamma (IFNγ), a key cytokine that influences the outcome of *Chlamydia* infections. At the concentrations at which CPE2_0552 inhibited T lymphocyte proliferation and IFNγ secretion, negligible cytotoxicity was observed after 72 h of stimulation. Our study indicates that *E. coli* lymphostatin belongs to a wider family of lymphocyte-inhibitory molecules that exist in distantly related bacterial pathogens.

## Introduction

Bacterial pathogens have evolved diverse strategies to evade or inhibit innate, humoral and cell-mediated responses of their hosts during infection. Examples of bacterial virulence factors that interfere with lymphocyte function are relatively scarce but include superantigens that drive clonal deletion and anergy of T cells, enzymes that interfere with the metabolic programming of T cells, and factors that inhibit lymphocyte chemotaxis or induce apoptosis [reviewed in [1]]. Another strategy used by some bacterial pathogens is to secrete proteins that interfere with the activation of lymphocytes and their signalling, with a notable example being lymphostatin from attaching and effacing *Escherichia coli*.

Lymphostatin (also known as lymphocyte inhibitory factor A; LifA) was first identified in the enteropathogenic *E. coli* (EPEC) strain E2348/69 [2]. EPEC products have been reported to inhibit the mitogen-activated proliferation of lymphocytes derived from the blood, spleen and intestines [3–5]. Screening of a library of cosmid clones for inhibitory activity and mutation of the *lifA* gene identified lymphostatin as the factor responsible [2]. LifA is a 365 KDa protein that exhibits N-terminal homology with the glycosyltransferase domain of large clostridial toxins (LCTs) [2]. LCTs act by glycosylating Rho-family GTPases that regulate the actin cytoskeleton and other cellular processes [reviewed in [6]]. Highly purified LifA has been reported to inhibit mitogen- and antigen-activated proliferation of bovine T lymphocytes in the femtomolar range [7]. It inhibits the CD4, CD8, WC-1 and γδ T cell receptor subsets of T lymphocytes and renders them refractory to mitogenic stimulation for at least 18 h after transient exposure [8]. Lymphostatin also inhibits the activity of bovine natural killer cell and B lymphocytes to a lesser extent, as well as the production of interleukin (IL)-2, IL-4, IL-10, IL-17A and interferon γ by mitogen-activated T cells [8]. Similar activity has been reported against human T lymphocytes, together with evidence that lymphostatin arrests the cell cycle at the G1 phase but does not cause apoptosis or necrosis [9].

Lymphostatin activity depends on a DXD motif in the putative glycosyltransferase domain [7,9] that is necessary for the binding of uridine diphosphate N-acetylglucosamine (UDP-GlcNAc) [7]. It has been inferred, but not proven, that lymphostatin acts by transferring GlcNAc onto one or more cellular targets. Lymphostatin also shares a cysteine protease motif with LCTs and YopT superfamily proteins [10]. In LCTs, the cysteine protease domain mediates autocatalytic cleavage following a low pH-induced conformational change that leads to the insertion of the protein through the endosomal membrane [6]. This cleavage event releases the N-terminal glycosyltransferase domain of LCTs into the cytosol. Consistent with a similar role in LifA processing, a C1480A substitution in the cysteine protease domain abolished lymphostatin activity and the production of a 140 KDa N-terminal fragment containing the glycosyltransferase domain of LifA in treated cells [11].

Lymphostatin plays an important role in intestinal colonization by the murine attaching and effacing pathogen *Citrobacter rodentium* in mice [12] and Shiga toxin-producing *E. coli* (STEC) serogroup O5, O26 and O111 in calves [13,14]. The extent to which this is attributable to its activity against lymphocytes, as opposed to its influence on adherence to host cells [15], and indirect effects on Type 3 secretion in some strains [13,14], is unclear. LifA has also been reported to be secreted via the Type 3 secretion system in EPEC [16], and to influence actin nucleation at sites of attachment to human intestinal explants [17]. Injection of LifA into host cells via the Type 3 secretion system is not necessary for lymphostatin activity, as such activity can be observed with bacterial lysates [2–5] and purified protein [7].

Lymphostatin homologues are present in other bacteria. STEC serotype O157:H7 strains lack LifA, but possess a pO157-encoded homologue termed ToxB [18]. At the amino acid sequence level, ToxB exhibits 29% identity and 62% similarity to LifA, and shares glycosyltransferase and cysteine protease domains. Affinity-purified ToxB inhibits the mitogen-activated proliferation of bovine T cells, albeit to a lesser extent than LifA [8]. Complete and partial homologues of lymphostatin have also been described in *Chlamydia trachomatis*, an obligate intracellular pathogen that causes ocular and urogenital diseases in humans [19]. These genes are encoded in a highly polymorphic region of *Chlamydia* genomes termed the plasticity zone (PZ) and selected genes from this region are associated with cytotoxicity [19]. Moreover, different cytotoxin loci in the PZ region are associated with non-invasive oculotropic, urogenitotropic, and invasive serotypes of *C. trachomatis* [20]. Only non-invasive urogenitotropic serotypes possess a putative cytotoxin with an intact N-terminal glycosyltransferase domain and a predicted UDP-glucose binding motif shared with LCTs [21]. One of the PZ-encoded cytotoxins in C. *trachomatis* serogroup D (CP166) disrupts the actin cytoskeleton via glucosylation of Rac [21], in a manner dependent on the DXD motif in its glycosyltransferase domain [22].

Sequence diversity in the cytotoxin-encoding PZ region has also been reported among isolates of *Chlamydia pecorum*, a significant pathogen of farmed animals (especially cattle, sheep, goats, and pigs), which also causes debilitating ocular and urogenital infections in koala [23–25]. *C. pecorum* infections in farmed animals manifest in diverse forms including polyarthritis, metritis, gastroenteritis, encephalomyelitis, mastitis and pneumonia. Genome sequencing of *C. pecorum* strains associated with polyarthritis, metritis, and inapparent enteric infection revealed that all three strains encoded two LCT homologues in the PZ region, although these varied in sequence and phylogeny [23]. Owing to their similarity with lymphostatin, the authors of this report speculated that these proteins may play a role in pathogenesis, possibly by modulating interferon γ synthesis as this is critical in determining the outcome of *Chlamydia* infection [reviewed in [26]]. To begin to dissect the role of PZ-encoded lymphostatin homologues, we cloned, purified and characterized one of these, CPE2_0552 from the W73 strain of *C. pecorum*.

## Methods

### Cloning strategy and sequence validation

The full-length (10,095 bp) *cpe2_0552* gene of *C. pecorum* was amplified with Phusion proofreading DNA polymerase (Thermo Fisher Scientific, Loughborough, UK) using the primers CP_FOR, 5′-GAAGGAATACATATGACTCTGCCGGAAGCGCCT-3′ and CP_REV, 5′-GTGATGGTGGTGATGATGAGTTTTTATAGGTAGCGTTAT-3′ and genomic DNA from strain W73, a genome-sequenced strain isolated in 1989 from the faeces of a sheep with asymptomatic infection [23] supplied by Professor David Longbottom, Moredun Research Institute. Following agarose gel electrophoresis an amplicon of the expected size was excised from the gel and purified using Geneclean II (MP Biomedicals, Irvine, USA). This was then cloned using the Lucigen Expresso® Rhamnose cloning and expression system (LGC Biosearch Technologies, Teddington, UK), as described for EPEC E2348/69 *lifA* [7]. This involved homologous recombination with linearized pRham C-His to generate an in-frame C-terminal fusion to a vector-encoded 6 × His tag to enable affinity purification, where CPE2_0552 expression was induced by rhamnose when required and repressed by glucose during cloning and passage. Putative recombinants were obtained by transformation of chemically competent *E. cloni* 10 G® cells (LGC Biosearch Technologies, Teddington, UK) and screened by colony PCR using primers flanking the insertion site in the pRham C-His plasmid (pRham Forward, 5′-GCTTTTTAGACTGGTCGTAGGGAG-3′ and pETite Reverse, 5′-CTCAAGACCCGTTTAGAGGC-3′). The sequence of one clone (pRham-*cpe2*_*0552*) was confirmed to be identical to the published *cpe2_0552* sequence of *C. pecorum* strain W73 [23] by full-length Sanger sequencing using primers every 500 bp on the forward strand (GATC Biotech, Wolverhampton, UK).

### Bioinformatic analysis of CPE2_0552

The amino acid sequences of CPE2_0552 from *C. pecorum* strain W73 (accession number AGW38961) and LifA from EPEC strain E2348/69 (accession number WP_001239084) were aligned with T-Coffee version 11.00 using default parameters [27]. Partial alignments spanning the DXD and LNG motifs in the glycosyltransferase domain and the CHD catalytic triad in the cysteine protease domain were obtained using PSI-Coffee using default parameters [28]. To highlight regions of local similarity, we performed a pair-wise alignment of the two sequences using LALIGN (BLOSSUM62 matrix [29]), visualized in LALNVIEW [30]. The following amino acid sequences were used for this analysis: *Chlamydia pecorum* CPE2_0552 (accession number AGW38961), *Chlamydia muridarum* LifA/Efa1-related large cytotoxin (accession number WP_010904337), *Chlamydia trachomatis* hypothetical protein CT166 (accession number ABA42639), *Chlamydia felis* LifA/Efa1-related large cytotoxin (accession number WP_011457994), *Chlamydia caviae* LifA/Efa1-related large cytotoxin (accession number WP_011006516), *Chlamydia suis* LifA/Efa1-related large cytotoxin (accession number WP_080141411), *Chlamydia gallinacea* LifA/Efa1-related large cytotoxin (accession number WP_338077760), *Chlamydia ibidis* LifA/Efa1-related large cytotoxin (accession number WP_021119564), *Chlamydia psittaci* LifA/Efa1-related large cytotoxin (accession number WP_279492445), *Providencia alcalifaciens* DUF3491 domain-containing protein (accession number WP_051420264), *Escherichia albertii* lymphostatin Efa1/LifA (accession number WP_000701120), *Citrobacter rodentium* DUF3491 domain-containing protein (accession number WP_012907284), *Escherichia coli* O127:H6 lymphostatin Efa1/LifA (accession number WP_001239084), *Escherichia coli* O157:H7 putative cytotoxin (accession number AAC70163); *Grimontia hollisae* TcdA/TcdB catalytic glycosyltransferase domain-containing protein (accession number WP_115660322), *Escherichia coli* O127:H6 Efa1/LifA-like (accession number CAS08627), *Shigella dysenteriae* toxin (accession number EGE2519152), *Clostridioides difficile* glycosylating toxin TcdB (accession number WP_332969363).

AlphaFold 3 [31] was used to predict the tertiary structure of full-length LifA, and the predicted glycosyltransferase domain (residues 237–771 of strain E2348/69 LifA) and cysteine protease domain (residues 1442–1653 of LifA) relative to the homologous regions in CPE2_0552 from strain W73 CPE2_0552 (residues 260–862 and 1546–1758, respectively). Predicted structural models were generated using an automated seed with default parameters and the highest-ranking structure was visualized using PyMOL Molecular Graphics Systems, version 3.0.3 Schrödinger, LLC. Predicted template modelling (pTM) scores were presented as a measure of superposition between the predicted structure and hypothetical true structure. pTM scores vary between 0 and 1, where a score above 0.5 means the overall predicted fold for the complex will be similar to the true structure.

### Expression and purification of *C.* pecorum W73 CPE2_0552 and *E.* *coli* E2348/69 LifA

Recombinant 6 × His-tagged *E. coli* E2348/69 LifA was expressed in *E. cloni*® cells (Cambridge Biosciences, Cambridge, UK), as previously described [7]. W73 CPE2_0552 from *C. pecorum* was expressed and purified as follows. Transformed cells (Rosetta™ 2 (DE3) cells; Novagen, Madison, USA) from a single colony were cultured in lysogeny broth containing 30 µM kanamycin and 34 µM chloramphenicol at 30°C, 250 and rpm for 16 h. A 1/200 inoculum was then transferred to 500 mL lysogeny broth with the same antibiotics in a 2 L baffled Erlenmeyer flask. This was incubated at 37°C, 250 rpm shaking to A_600_ nm of 0.4. Flasks were cooled to 30°C and expression of the protein was induced by the addition of 0.2% (w/v) L-rhamnose and cultured for a further 3 h. Cells were pelleted by centrifugation and resuspended in 25 mL BugBuster Master Mix reagent (Novagen, Madison, USA) per litre of the original volume. This was supplemented with ethylenediaminetetraacetic acid (EDTA)-free protease inhibitor cocktail tablets (Roche, Welwyn Garden City, UK) and 100 µM phenylmethanesulfonyl fluoride (PMSF) and incubated with gentle agitation for 1 h at 4°C. The lysate was then sonicated for six cycles of 10 s on ice. All chromatography was performed on an ÄKTAPURE protein purification system (Cytiva, Little Chalfont, UK) at 8°C. The cell lysate was clarified by centrifugation at 50,000 × *g*, at 4°C for 1 h and the supernatant was filtered through a 0.2 µm filter. The clarified supernatant was then loaded onto 1 mL HiTrap IMAC FF column charged with Ni^2+^ ions (Cytiva, Little Chalfont, UK) pre-equilibrated in *Buffer A* (comprising 20 mm sodium phosphate, 300 mm sodium chloride, 10% (v/v) glycerol, 1 mm dithiothreitol (DTT), 0.1% (v/v) Tween 20, 0.1% (v/v) Triton X-100 reduced, 1% (w/v) CHAPS, 10 mm imidazole, pH 7.8), at 1 mL/min. This was followed by washing with 15 column volumes (cv) of *Buffer A* and 15 cv of *Buffer A* supplemented with 30 mm imidazole. CPE2_0552 was eluted by addition of *Buffer B* (20 mm sodium phosphate, 300 mm sodium chloride, 500 mm imidazole, 10% (v/v) glycerol, 1 mm DTT, 0.1% (v/v) Tween 20, 0.1% (v/v) Triton X-100 reduced, 1% (w/v) CHAPS, pH 7.8).

Fractions containing CPE2_0552 were concentrated using a Vivaspin® molecular weight cut-off 100 KDa filter (Sartorius, Epsom, UK) and loaded onto a Superose-6 10/300 GL (Cytiva, Little Chalfont, UK) gel filtration column pre-equilibrated in *Buffer C* (20 mm sodium phosphate, 300 mm sodium chloride, 10% (v/v) glycerol, 1 mm DTT, 0.1% (v/v) Tween 20, 0.1% (v/v) Triton X-100 reduced, pH 7.8) and run at 0.5 mL/min. Fractions containing CPE2_0552 were concentrated and reapplied to the same gel filtration column, pre-equilibrated in 10 mm Tris, 300 mm NaCl, 0.1 mm DTT, pH7.8. CPE2_0552 eluted at 13.25 mL. CPE2_552 was then concentrated to 1 mg/mL, supplemented with 20% (v/v) glycerol, flash frozen in liquid nitrogen, and stored in aliquots at −80°C. Purified proteins were analyzed by 3–8% Tris Acetate sodium dodecyl sulphate-polyacrylamide gel electrophoresis (SDS-PAGE) and Coomassie Brilliant Blue staining.

### Negative staining electron microscopy and image processing

Purified CPE2_0552 (4 µL at a concentration of 20 µg/mL) was applied to freshly glow-discharged 300 mesh copper-grids with continuous carbon-film (C267, TAAB) and incubated for 2 min. Grids were then washed with two drops of water and two drops of 2% (w/v) uranyl acetate followed by staining with a further drop of 2% (w/v) uranyl acetate for 2 min. The excess liquid was blotted from the edge of the grid using filter paper. The grids were loaded in a Thermo Fisher Scientific F20 transmission electron microscope operated at 200kV. Images were collected manually using EMMENU software (Tietz Video & Imaging Processing Systems (TVIPS), Gilching, Germany) on a TemCam F816 camera (TVIPS), using 8kb2 mode, a magnification of 100,000 × corresponding to a calibrated pixel size of 1.48 Å/pixel. Data were collected with a total applied dose of 60–70 electrons/Å^2^ and a defocus range of −0.5 to −1.5 µm.

Electron micrographs were imported into RELION 3.1.0 [32], and the contrast transfer function was estimated using Gctf [33]. Micrographs with poor drift characteristics, were discarded after manual inspection using RELION software. Particle picking was performed with EMAN2 [34] using a neural net trained by manually selecting particles from the data. From these particle coordinates 34,804 particles were extracted in RELION and subjected to 2D classification, until 9,240 particles remained in the final 2D-classes. From these particles, one 3D volume was obtained using the 3D classification in RELION 3.1.0.

### Isolation of T cells from bovine blood

Venous blood was collected from three healthy 12- to 18-month-old Holstein-Friesian cows housed at the Dryden Farm of the University of Edinburgh in full compliance with the requirements of the Animals (Scientific Procedures) Act 1986 under the Home Office project licence P803DD07A, with the approval of the Animal Welfare and Ethical Review Board of The Roslin Institute and in accordance with ARRIVE guidelines. Following centrifugation at 1,200 × *g* for 15 min, white blood cell fractions were collected and layered over Ficoll-Paque Plus (GE Healthcare, Amersham, UK) and centrifuged for 30 min at 1,200 × *g* with no brake. The interface layer containing peripheral blood monocytes was collected and washed three times with sterile phosphate-buffered saline (PBS; Oxoid, Basingstoke, UK). The T lymphocyte fraction was further enriched using a sterile nylon wool column (Polysciences, SciQuip, Wem, UK). Columns were washed twice with sterile RPMI-1640 medium supplemented with 10% (v/v) foetal bovine serum, 20 mm HEPES, 1 mm sodium pyruvate, 100 units/mL of penicillin and streptomycin, 20 mm L-glutamine (all from Life Technologies, Paisley, UK), and then incubated for 1 h at 37°C in a 5% CO_2_ atmosphere, using two volumes of the same medium. Cells were resuspended in the same medium, applied to the column at 10^8^/mL and incubated for 1 h at 37°C, 5% CO_2_. Unbound cells (mainly T cells) were washed off the column in 10 mL of medium, collected by centrifugation at 1,200 × *g*, resuspended, and counted. The purity of the T cell preparations was analysed by single channel flow cytometry. Cells were stained with an anti-bovine CD3 antibody (MM1A; IgG1; Bio-Rad, Watford, UK), followed by staining with a fluorescein isothiocyanate (FITC)-conjugated anti-IgG1 secondary antibody. The samples were analysed on a Fortessa X20 flow cytometer using CellQuest with FlowJo™ v10 software (BD Biosciences, Wokingham, UK). A minimum of 10,000 events were collected, with an initial gate for live cells based on the forward/side scatter parameters. The purity of final T cell preparations was > 85% in each replicate, consistent with earlier studies [8].

### Proliferation assay

Enriched T cells were used to test the activity of affinity-purified CPE2_0552 or LifA using a colorimetric assay for mitogen-induced proliferation, as previously described [7]. Cells were plated at a density of 2 × 10^5^ cells/well in 96-well flat-bottom plates in triplicate for all conditions. CPE2_0552 or LifA were added to the final concentrations indicated in the figures. Medium-only and cell-only negative controls were used. Cell proliferation was stimulated using the mitogen concanavalin A (ConA; Merck, Glasgow, UK) at a final concentration of 0.5 μg/mL in the presence or absence of recombinant CPE2_0552 or LifA, in a final volume of 100 μL/well. Th cells were then incubated at 37°C for 72 h. CellTiter 96® Aqueous One substrate (Promega, Southampton, UK) was added 18 h before the end of the assay. Absorbance was measured at 492 nm using a Multiskan Ascent plate reader (Thermo Fisher Scientific, Loughborough, UK). The background medium measurements were subtracted from all values. The Proliferation Index was calculated by dividing the absorbance reading for cells treated with ConA and recombinant protein by the absorbance reading for cells treated with ConA alone. A Proliferation Index of 1 indicates no inhibition of ConA-stimulated proliferation whereas values below 1 indicated inhibition. ConA-stimulated responses were typically 2-fold higher than those of unstimulated cells in each replicate. The carrier buffer for both recombinant proteins was determined to have no effect on the ConA stimulation of cells. The dose at which 50% inhibition of proliferation was detected (ID_50_) for CPE2_0552 and LifA were determined using the drm function of the drc package [35] in R (R Foundation for Statistical Computing, Vienna, Austria).

### Measurement of IFNγ secretion by T cells

ConA-activated T cell proliferation assays were performed as described above in the presence of CPE2_0552, LifA or the diluent control. Culture supernatants were collected after 24 h and centrifuged at 1200 × *g* for 5 min. An enzyme-linked immunosorbent assay (ELISA) was used to measure the IFNγ concentration in the supernatants. Briefly, Nunc Immunosorb 96-well plates (Thermo Fisher Scientific, Loughborough, UK) were coated with the anti-bovine IFNγ capture antibody CC330 (Bio-Rad, Watford, UK) and incubated at 4°C overnight. Plates were washed five times with wash buffer (PBS with 0.05% (v/v) Tween 20) and blocked for 1 h at room temperature in PBS containing 1 mg/mL of sodium casein. Plates were washed five times in wash buffer and then the undiluted supernatants were added. The plates were incubated for 1 h at room temperature, washed again five times with wash buffer, and mouse anti-bovine IFNγ detection antibody CC302b was added (Bio-Rad, Watford, UK). Plates were incubated for 1 h, washed five times in wash buffer and anti-mouse Ig streptavidin-horseradish peroxidase conjugate (Bio-Rad, Watford, UK) was added. The plates were incubated for 1 h and washed a final five times with wash buffer. The signal was developed using a 3,3′,5,5′-tetramethylbenzidine substrate solution (BioLegend, London, UK). Optical density was measured at 450 nm using a Multiskan Ascent plate reader.

### Cytotoxicity assay

The cytotoxic effects of CPE2_0552 and LifA were determined using a Cytotoxicity Detection Test Plus assay for the detection of released lactate dehydrogenase (LDH; Roche, Welwyn Garden City, UK) according to the manufacturer’s instructions. LDH release was analysed at 24 and 72 h after addition of the mitogen and proteins as described for the proliferation assays. The background values for the cell-only control were subtracted from all other values. A control in which cells were lysed at the time of reading the assay was included to quantify maximal LDH release. Data are expressed as percentage cytotoxicity, calculated as follows: (absorbance of experimental condition – absorbance for cell only control)/(absorbance for lysed control – absorbance for cell-only control) × 100.

### Statistical analysis

The significance of differences in lymphocyte proliferation, IFNγ production and cytotoxicity between LifA- or CPE2_0552-treated cells and ConA- and buffer-treated controls were analysed by *t* tests. *p* values ≤ 0.05 were taken to be significant.

## Results

### *Chlamydia pecorum* strain W73 CPE2_0552 exhibits significant homology with lymphostatin

**Figure 1.**
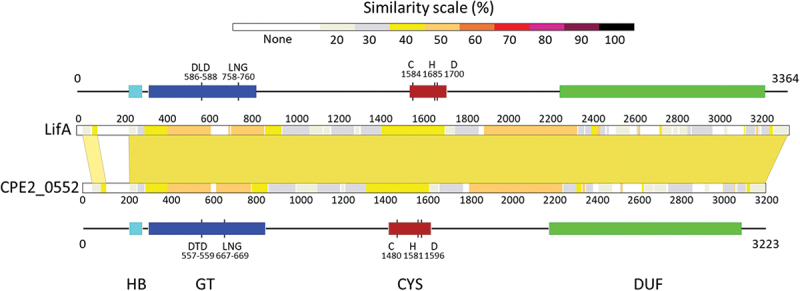
Regions of amino acid sequence similarity between CPE2_0552 and lymphostatin.

In *C. pecorum* strain W73, CPE2_0552 was predicted to be a 3364 amino acid 382 KDa protein. It exhibits 36% identity and 55% similarity over 3171 amino acids to LifA from EPEC strain E2348/69, in which lymphostatin was first described [2]. The extent of similarity was higher in the predicted functional domains (Figure 1). As with lymphostatin, CPE2_0552 lacks a signal peptide for the Sec translocon.

**Figure 2.**
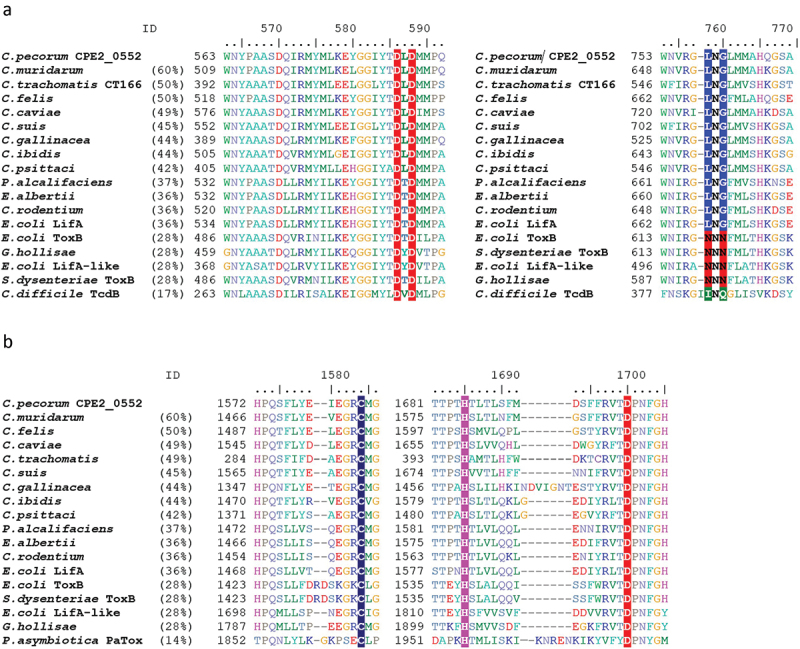
CPE2_0552 shares predicted catalytic motifs with lymphostatin.

CPE2_0552 shares an N-terminal glycosyltransferase domain with LifA, including the DXD motif required for lymphostatin activity and UDP-GlcNAc binding [7] (Figure 2a; DLD_586–588_ in CPE2_0522 and DTD_557–559_ in LifA). Within this domain, CPE2_0552 also shares an LNG motif (residues 758–760) with LifA (residues 667–669), which by analogy with LCTs, is assumed to bind to a UDP-sugar donor molecule in proximity to the active site. CPE2_0552 also shares a cysteine protease domain from the YopT superfamily (C58 class), with conservation of the predicted catalytic triad (residues C_1584_, H_1685_, D_1700_) relative to lymphostatin (C_1480_, H_1581_, D_1596_)(Figure 2b). Our phylogenetic analysis of the cysteine protease domain has previously shown that CPE2_0552 and LifA are clustered together [11]. In LifA, substitution of C1480 with alanine abolished lymphostatin activity and the appearance of an N-terminal 140 KDa fragment in T cells, inferred to be the product of autocatalytic cleavage. Both proteins also share homology in a large C-terminal domain of unknown function (DUF3491), presumed from studies on LCTs to be involved in receptor binding and translocation across the endosomal membrane.

Partial sequence alignment of CPE2_0552 (top row), lymphostatin from E2348/69 (*E. coli* LifA) and lymphostatin homologues from pathogenic bacteria (see Methods), focused on DXD and LNG motifs within the glycosyltransferase domain (panel A) and CHD catalytic triad within the cysteine protease domain (panel B). Sequences are ordered based on overall percentage identity to CPE2_0552.

**Figure 3.**
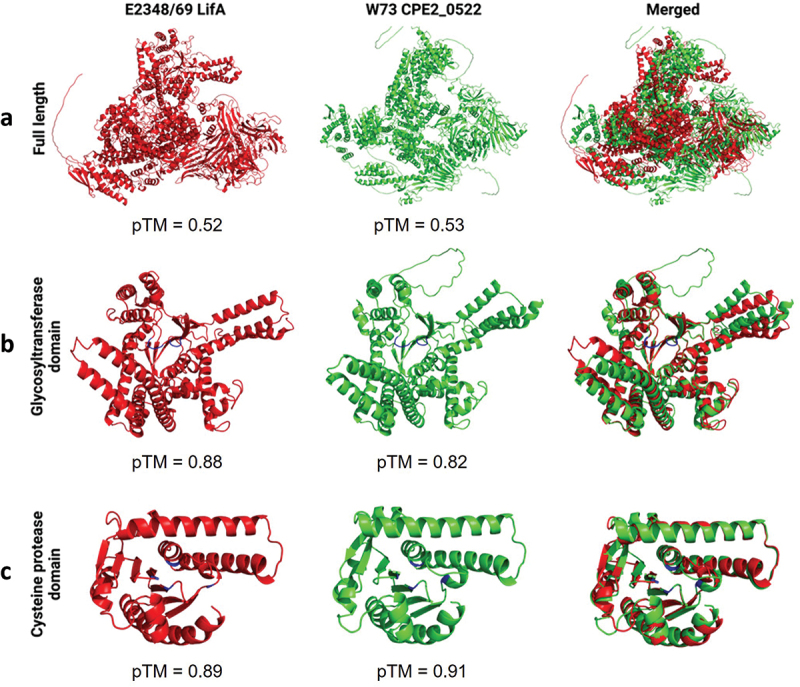
AlphaFold 3 predictions of the tertiary structure of full-length LifA from *E. coli* E2348/69 and CPE2_0522 from *C. pecorum* W73 (a), and the predicted glycosyltransferase domain (b) and cysteine protease domain (c). The location of the predicted active site in the glycosyltransferase and cysteine protease domains is marked in blue.

AlphaFold 3 was used to predict the tertiary structure of full-length LifA and CPE2_0552, as well as their glycosyltransferase and cysteine protease domains. While predictions for the full-length proteins have relatively low confidence (Figure 3a; pTM values of 0.52 and 0.53 for E2348/69 LifA and W73 CPE2_0522 respectively), those for the glycosyltransferase domain (Figure 3b; pTM values of 0.88 and 0.82 for E2348/69 LifA and W73 CPE2_0522, respectively), and cysteine protease domain (Figure 3c; pTM values of 0.89 and 0.91 for E2348/69 LifA and W73 CPE2_0522, respectively) have high confidence. Merging the predicted structures showed a high level of predicted structural similarity in the regions containing the predicted catalytic site (Figures 3b,c).

### Affinity purification of CPE2_0552 and lymphostatin

**Figure 4.**
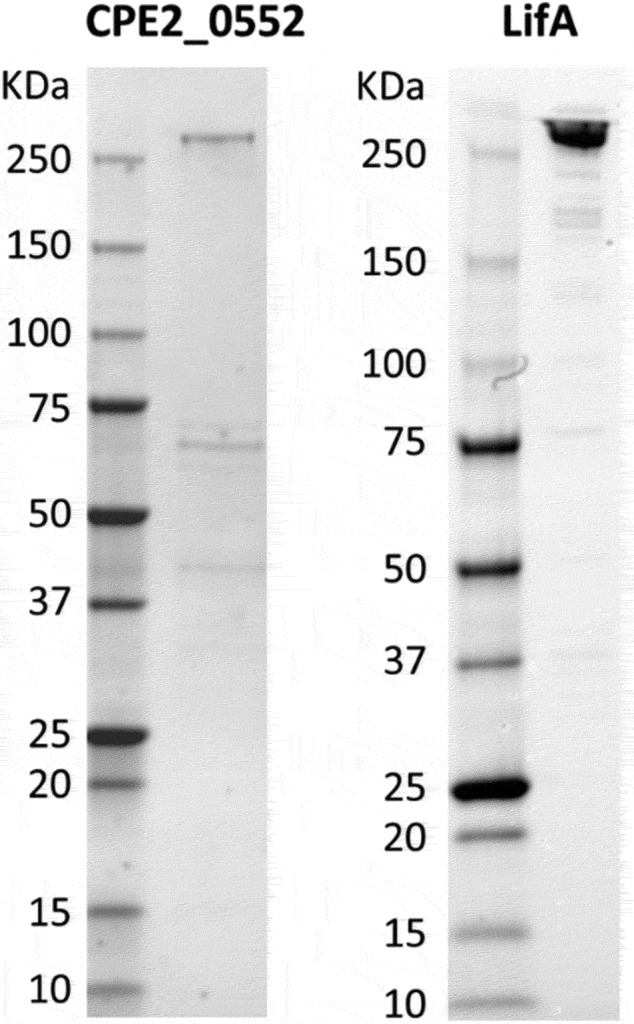
SDS-PAGE analysis of purified CPE2_0552 and LifA.

*C. pecorum* strain W73 CPE2_0552 and EPEC strain E2348/69 LifA with C-terminal 6 × his tags were purified using Ni^2+^ affinity chromatography followed by size separation as described in the Methods section. Proteins of the expected size were visualized by 3–8% Tris acetate SDS-PAGE (Figure 4). The yield of CPE2_0552 was markedly lower.

Proteins were stained with Coomassie Brilliant Blue. Original un-cropped images of the gels can be accessed as described in the Data Availability Statement.

### Surface envelope of CPE2_0552

**Figure 5.**
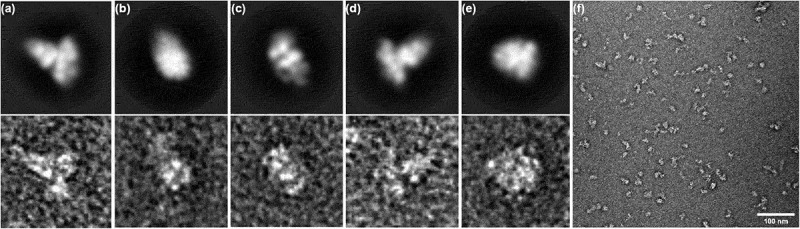
Representative transmission electron micrographs of negatively stained CPE2_0552 particles in different orientations. Panels A-E show 2D classes of particles (top row) and representative original images (bottom row) from five classes. Panel F shows an overview of particles, confirming they are monomeric and dispersed (scale bar 100 nm).

**Figure 6.**
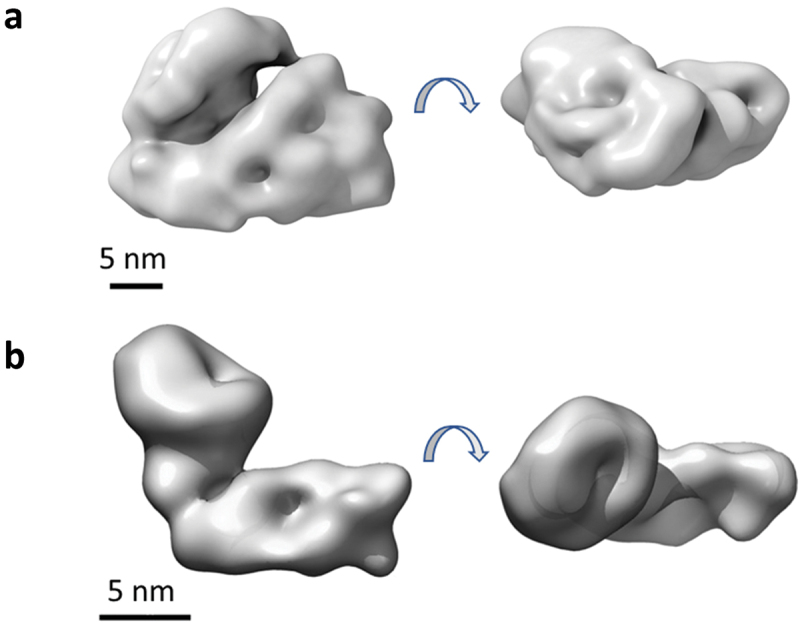
Predicted 3D surface representation and volume of CPE2_0552 (panel A). Our previously reported surface representation of lymphostatin [7] is shown for comparison (panel B).

The globular structure of CPE2_0552 was analyzed by transmission electron microscopy. Negatively stained CPE2_0552 showed a homogeneous distribution of monomeric particles (Figure 5). Images of 34,804 particles were extracted and subjected to 2D classification until 9,240 particles remained in the final 2D classes of five types (Figure 5). Based on these results, the 3D volume of CPE2_0552 was estimated (Figure 6). The surface representation previously reported for LifA [7] is shown for comparison (Figure 6). CPE2_0552 does not share the L-shaped globular structure observed for lymphostatin by transmission electron microscopy and small angle X-ray scattering [7,11].

### CPE_0552 inhibits the mitogen-activated proliferation of bovine T cells

**Figure 7.**
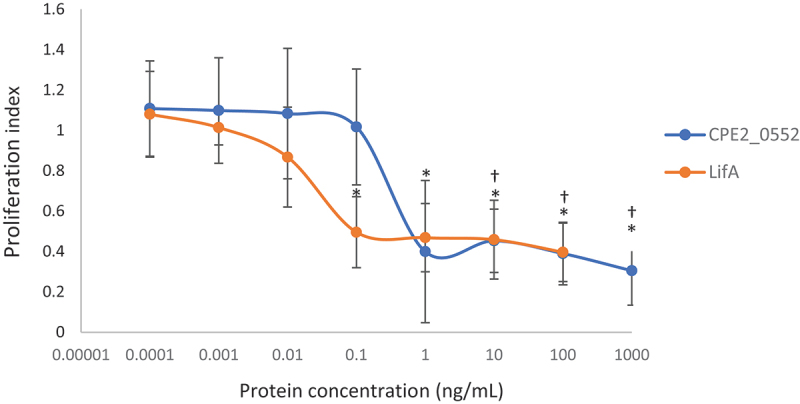
Concentration-dependent inhibition of T cell proliferation by CPE2_0552 and LifA.

As lymphostatin inhibits the mitogen-activated proliferation of bovine lymphocytes, we sought to determine if CPE2_0552 shares this activity. Enriched bovine T cells were obtained from three independent donors. Purified CPE2_0552 inhibited the ConA-induced proliferation of T cells in the picomolar range, with concentration-dependent titration of activity across a sigmoidal curve (Figure 7). The inhibitory dose at which 50% inhibition of proliferation was detected (ID_50_) were calculated for CPE2_0552 to be 812 ± 668 pg/mL and for LifA to be 21 ± 10 pg/mL, making CPE2_0552 38.7-fold less potent than LifA under the assay conditions. The ID_50_ for LifA was consistent with that for separate batches of the protein tested previously (14 ± 1.5 pg/mL in [7], 13 ± 10 pg/mL in [8], and 14 ± 4 pg/mL in [11]).

The data show the inhibitory effect of varying concentrations of *C. pecorum* strain W73 CPE2_0552 on ConA-induced proliferation of peripheral bovine T cells, compared to the effect of EPEC strain E2348/69 LifA. Concentrations on the *x* axis are displayed on a logarithmic scale. The data are the average of 3 independent donors, each performed in triplicate. Error bars indicate the standard deviation. Treated cells from the same experiments were used to assay IFNγ secretion and cytotoxicity. Similar sigmoidal curves showing concentration-dependent inhibition by LifA and CPE2_0552 were obtained for T cells from the three separate donors and original data can be accessed as described in the Data Availability Statement. *p* values < 0.05 for differences relative to the ConA- and buffer-treated control are indicated by * for LifA and † for CPE2_0552.

### CPE2_0552 inhibits IFNγ secretion from mitogen-activated T cells

**Figure 8.**
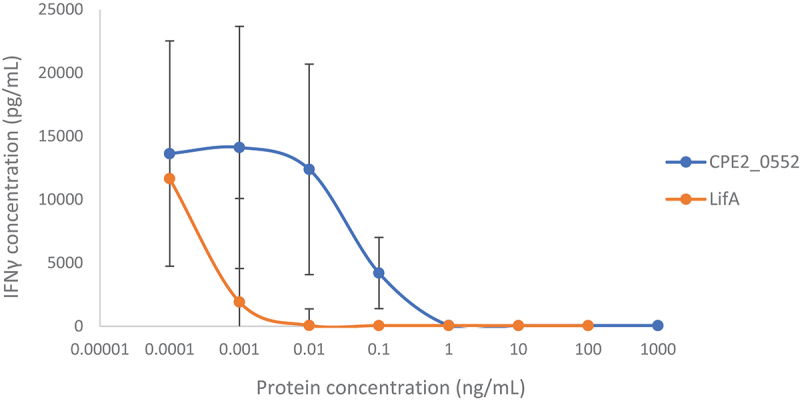
Concentration-dependent inhibition of IFNγsecretion from T cells by CPE2_0552.

Data collected using cells from the same donors, in parallel with assays of mitogen-activated proliferation and cytotoxicity, indicated that CPE2_0552 was capable of inhibiting the secretion of IFNγin a concentration-dependent manner (Figure 8). Secretion of IFNγ was fully inhibited by CPE2_0552 at a concentration of 1 ng/mL, at which inhibition of cellular proliferation was apparent but not maximal. At concentrations of 10 ng/mL and higher, both cellular proliferation and IFNγsecretion were fully inhibited (Figure 8). The concentration of lymphostatin required to fully inhibit ConA-activated IFNγ secretion by bovine T cells was lower than that required for CPE2_0552 and was comparable to that reported previously [7]. While differences relative to ConA-treated cells did not reach statistical significance owing to inter-animal variance, it is biologically significant that the reduction in IFNγ detected was concentration dependent and virtually no IFNγ was detected at concentrations at which lymphocyte proliferation was inhibited.

The data show the inhibitory effect of varying concentrations of *C. pecorum* strain W73 CPE2_0552 on ConA-activated proliferation of peripheral bovine T cells, compared to the effect of EPEC strain E2348/69 LifA. Concentrations on the *x* axis are displayed on a logarithmic scale. The data are the average of 3 independent donors, each performed in triplicate. Error bars indicate the standard deviation. Treated cells from the same experiments were used to assay ConA-stimulated proliferation and cytotoxicity. Similar curves showing concentration-dependent inhibition by LifA and CPE2_0552 were obtained for T cells from the three separate donors, and original data can be accessed as described in the Data Availability Statement.

### Inhibition of lymphocyte function by CPE2_0552 is not a consequence of direct cytotoxicity

**Figure 9.**
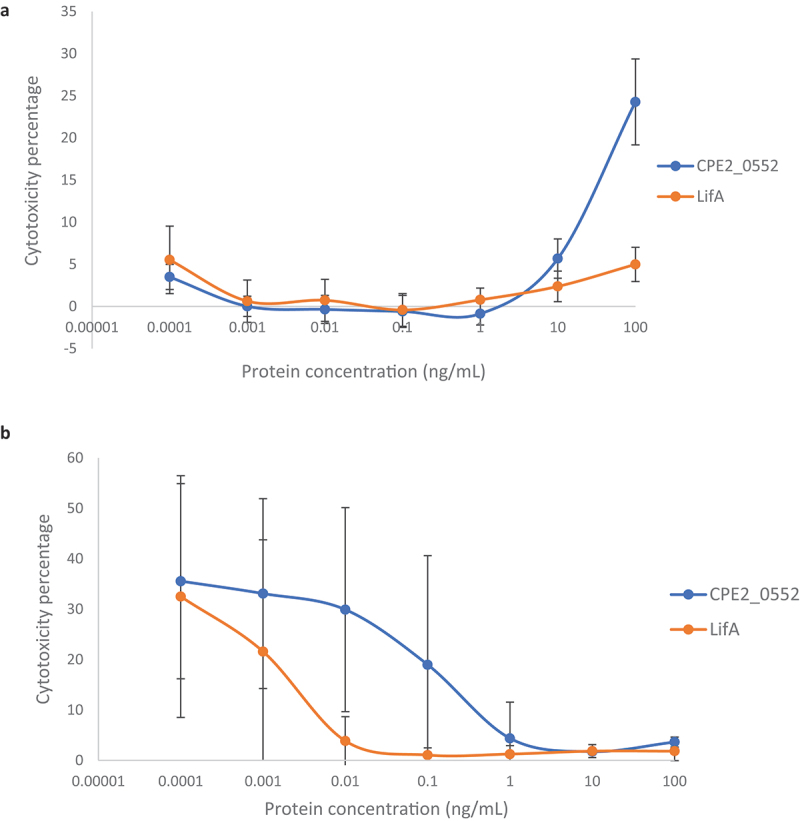
Inhibition of lymphocyte function by CPE2_0552 is not associated with cytotoxicity.

The release of cytosolic lactate dehydrogenase (LDH) into culture supernatants was quantified 24 h and 72 h after the addition of ConA and CPE2_0552 or LifA at a range of concentrations in the same assays used to measure proliferation and IFNγ secretion. After 24 h, a maximum cytotoxicity of 24.3% was detected after 24 h of exposure at the highest concentration of CPE2_0552 used (100 ng/mL). At a concentration of 10 ng/mL, at which there was maximal inhibition of ConA-stimulated proliferation by CPE2_0552, a cytotoxicity of 5.67% was detected, compared to 2.38% cytotoxicity for LifA at the same concentration (Figure 9a). After 72 h of exposure, negligible cytotoxicity was detected over the range of 1–100 ng/mL of CPE2_0552, at which inhibition of ConA-activated proliferation and IFNγ secretion was observed (Figure 9b). At these concentrations, cytotoxicity was not significantly different from the ConA- and buffer-treated control. Thus, the inhibition of lymphocyte function by CPE2_0552 at these concentrations is not associated with damage to the cells. As the concentrations of both proteins decreased, cytotoxicity detected at 72 h increased. This can be explained by the lack of protein to inhibit ConA-activated proliferation, such that the lymphocytes are driven to exhaustion and necrosis by the mitogen (Figure 9b).

Culture supernatants were used from the same assays as used to quantify ConA-activated T cell proliferation and IFNγ secretion over a range of concentrations of CPE2_0552 or LifA. Concentrations on the *x* axis are displayed on a logarithmic scale. Percentage cytotoxicity (calculated relative to the total amount of LDH released by cell lysis) was determined after 24 h (panel A) and 72 h (panel B). Concentrations on the *x* axis are displayed on a logarithmic scale. The data are the average of 3 independent donors, each performed in triplicate. Error bars indicate the standard deviation. Similar curves were obtained for T cells from the three separate donors treated with LifA or CPE2_0552 and original data can be accessed as described in the Data Availability Statement.

## Discussion

The plasticity zone in the genomes of pathogenic *Chlamydia* species is highly polymorphic and can encode complete and partial homologues of large clostridial toxins [19,20,23–25]. Variations in this region are associated with different outcomes of infection [20,23] and can distinguish strains associated with different host species [25]. *C. trachomatis* serotypes encoding one or more of these LCT-like proteins (MoPN and D), but not a serotype lacking them (L2), were cytotoxic to HeLa cells and collapsed the actin cytoskeleton [19]. One such protein (CT166) from a serotype D strain can cause this effect when ectopically expressed in HeLa cells in a manner that requires a DXD motif within its glycosyltransferase domain and which was associated with Rac1 glucosylation [21,22]. It is unclear whether CP166 retains this activity when applied exogenously to cells as it lacks the domain of LCTs required for uptake and membrane translocation in endosomes. Growth-independent cytotoxicity phenotypes have also been ascribed to three LCT homologues encoded in the *C. muridarum* PZ. However, the generation of chimeric strains by lateral gene transfer from *C. trachomatis* has indicated that other factors may be involved [36].

Not all LCT homologues in pathogenic bacteria appear to possess cytotoxic activities. Lymphostatin from attaching and effacing *E. coli* does not cause the release of cytosolic lactate dehydrogenase in treated T lymphocytes [7] and effects on the actin cytoskeleton after treatment of cells have not been reported. Rather, lymphostatin is a potent inhibitor of mitogen- and antigen-activated proliferation of lymphocytes and pro-inflammatory cytokine synthesis [2,7,8]. Such activity could be particularly significant during *Chlamydia* infection, where IFNγ plays a pivotal role in determining the outcome of infection [26]. At low IFNγ concentrations acute infections typically occur, whereas persistence and clearance of *Chlamydia* infection occurs at medium and high IFNγ concentrations, respectively, possibly through the depletion of host tryptophan needed for bacterial growth owing to induction of indoleamine 2,3-dioxygenase expression by IFNγ [37]. Sait *et al*. speculated that the ability of PZ-encoded cytotoxins (including CPE2_0552) to block IFNγ production may enable *C. pecorum* to cause acute disease and explain why strains that vary in the PZ region produce different clinical outcomes [23]. Phase variation has been reported to affect the function of CT166 in *C. trachomatis* isolates and it is plausible that *in vivo* regulation of PZ-encoded gene expression is used to modulate host responses [38]. The extent to which IFNγ affects *C. pecorum* is unclear, as studies have reported that *C. pecorum* is resistant to IFNγ in human and bovine cells, at least when exogenous tryptophan is supplied, unlike *C. trachomatis* which is inhibited under the same conditions [39]. Further studies are needed to understand if lymphostatin homologues in *Chlamydia* act in an additive way, and whether their absence in some strains is associated with the ability to synthesize tryptophan such that they are less sensitive to IFNγ-mediated control.

To address the hypothesis that CPE2_0552 possesses lymphostatin-like activity, we cloned, expressed and affinity-purified the protein from the genome of *C. pecorum* strain W73. The purified protein inhibited ConA-stimulated proliferation of enriched bovine T cells in a concentration-dependent manner, with an ID_50_ value of 812 ± 668 ng/mL. While this was 38.7-fold higher than the ID_50_ of *E. coli* E2348/69 lymphostatin tested in parallel on T cells from the same donors (21 ± 10 ng/mL) it was similar to that of ToxB, another LifA homologue from *E. coli* O157:H7 (1100 ± 880 pg/mL) [8]. We consider it unlikely that the inhibition observed with CPE2_0552 preparations is attributable to contaminants co-purified from the expression strain. We have previously shown that mutated forms of lymphostatin, affinity purified from the same *E. coli* strain in the same way, do not become inhibitory until much higher concentrations than those used for CPE2_0552. For example, the ID_50_ for a glycosyltransferase motif mutant of lymphostatin (D_557_-D_559_ to AAA substitution) was 922 ng/mL compared to 0.014 ng/mL for lymphostatin (65,857 higher [7]). Similarly, the ID_50_ for a cysteine protease motif mutant (C_1480_A substitution) was 1215 ng/mL compared to 0.014 ng/mL for lymphostatin (86,785 times higher [11]).

As lymphostatin is active against peripheral blood mononuclear cells from mice, cattle, and humans [2–7], and diverse bovine T cell subsets [8], it will be of interest to determine whether the activity of CPE2_0552 varies with animal species or cell populations. CPE2_0552 completely inhibited IFNγ secretion by ConA-activated bovine T cells at a concentration of 1 ng/mL. While CPE2_0552 was less inhibitory of IFNγ secretion than lymphostatin at lower concentrations, our data indicate that CPE2_0552 could block the production of a key cytokine governing *Chlamydia* pathogenesis and control. We cannot preclude the possibility that apparent differences in the magnitude of activity of CPE2_0552 and LifA may reflect differences in the folding, stability, or solubility of the recombinant proteins, or indeed differences in the substrates or co-factors required for their activity which may vary in abundance under assay conditions.

At concentrations where CPE2_0552 was able to block ConA-stimulated proliferation of bovine T cells and IFNγ secretion (e.g. 1 ng/mL to 100 ng/mL; Figure 7 and 8), we detected negligible cytotoxicity after 72 h of exposure based on the release of cytosolic lactate dehydrogenase (Figure 9). This suggests that its impact on lymphocyte function is not a consequence of direct cytotoxicity, as we have previously reported for lymphostatin using T cells from cattle [7] and humans [9]. Given the homology between CPE2_0552 and lymphostatin in the glycosyltransferase and cysteine protease domains, it is tempting to speculate that the activity of CPE2_0552 requires the DXD and CHD motifs, as we have described for LifA [7,11]; however, this will need to be analysed by site-directed mutagenesis. It is noteworthy that CPE2_0552 was significantly more challenging to express, purify and maintain in a stable and soluble form than LifA. Further optimization may be required to enable functional studies on CPE2_0552, for example to establish the role of specific motifs, study UDP sugar binding, and identify its interacting partner(s).

As lymphostatin is required for intestinal colonization by attaching and effacing pathogens [12–14], it is plausible that CPE2_0552 may also influence the virulence of *C. pecorum*. In a murine model of *C. muridarum* infection, the plasticity zone appears dispensable for colonization of the genital tract [40], as reinforced by the analysis of mutants lacking three genes encoding predicted cytotoxins in this region (*tc0437*, *tc0438*, and *tc0439*) [41]. However, the *tc0439* mutant (but not *tc0437* or *tc0438* mutants) was highly attenuated in the intestinal tract after oral or rectal inoculation [41], albeit this may be explained by secondary mutations detected by genome sequencing of the *tc0439* mutant [41]. Studies using defined mutants and repaired or complemented strains are required to formally establish the role of CPE2_0522 and other PZ-encoded LCT homologues in virulence. As observed with *lifA* mutants, it may be difficult to determine whether attenuation is due to inhibition of lymphocyte function, as indirect effects on other processes influencing colonization have been reported [13,14,17].

As *Chlamydia* is an obligate intracellular pathogen, it is important to consider how CPE2_0552 may come into contact with lymphocytes during infection. It is noteworthy that the cytotoxic activity of serotype MoPN and D strains of *C. trachomatis* associated with PZ-encoded proteins can be observed in the extracellular elementary body (EB) phase of development and does not require new transcription or translation [19], implying that EBs are charged with the proteins. The Type 3 secretion system is active in EBs [reviewed in [42]], and elementary bodies of *C. trachomatis* can be induced to secrete its effectors [43]. As LifA and LifA-like proteins are Type 3 secreted in EPEC [16], it would be interesting to study if CPE2_0552 is secreted via the Type 3 secretion system detected in the genome sequence of *C. pecorum* strain W73 [23]. An alternative strategy used by intra-vacuolar pathogens to secrete proteins is to hijack the biogenesis of vesicles released from the host cell [44]. Alternatively, they may hijack a cellular pathway for protein export, as has been reported for the typhoid toxin (CdtB) of *Salmonella* Typhi [45]. A simpler strategy may involve the release of CPE2_0552 from the infected cells upon lysis. Further studies using antibodies raised against the purified CPE2_0552 protein would be useful to define the location and timing of its expression and secretion, as well as to discern whether the protein binds to T cells and is processed within them, as observed for LifA [11]. Further, production of a CPE2_0552-specific antibody may permit analysis of the concentrations of the protein found during natural or experimental *C. pecorum* infections in order to set our findings using purified T cells *ex vivo* into context.

Our study expands the number of lymphostatin homologues that have been reported to inhibit lymphocyte proliferation and pro-inflammatory synthesis. Lateral gene transfer is likely to have played a significant role in their dissemination, as such homologues are frequently encoded on mobile elements or associated with highly polymorphic loci. The activities assigned to CPE2_0522 indicate that further research on predicted PZ-encoded cytotoxins is warranted, particularly to understand their role in host or niche adaptation and virulence in *Chlamydia* species.

## Supplementary Material

Figure S1A.jpg

Figure S1B.tif

## Data Availability

The data that support the findings of this study are available from the Data Share portal of the University of Edinburgh, via the following link: https://hdl.handle.net/10283/8839
