# Supplementary figures and images for: A lymphostatin homologue from Chlamydia pecorum inhibits mitogen-activated bovine T cell proliferation and IFNγ production
Source: Virulence. 2025 May 22;16(1):2506500. doi: 10.1080/21505594.2025.2506500 (PMC12118415; doi:10.1080/21505594.2025.2506500)

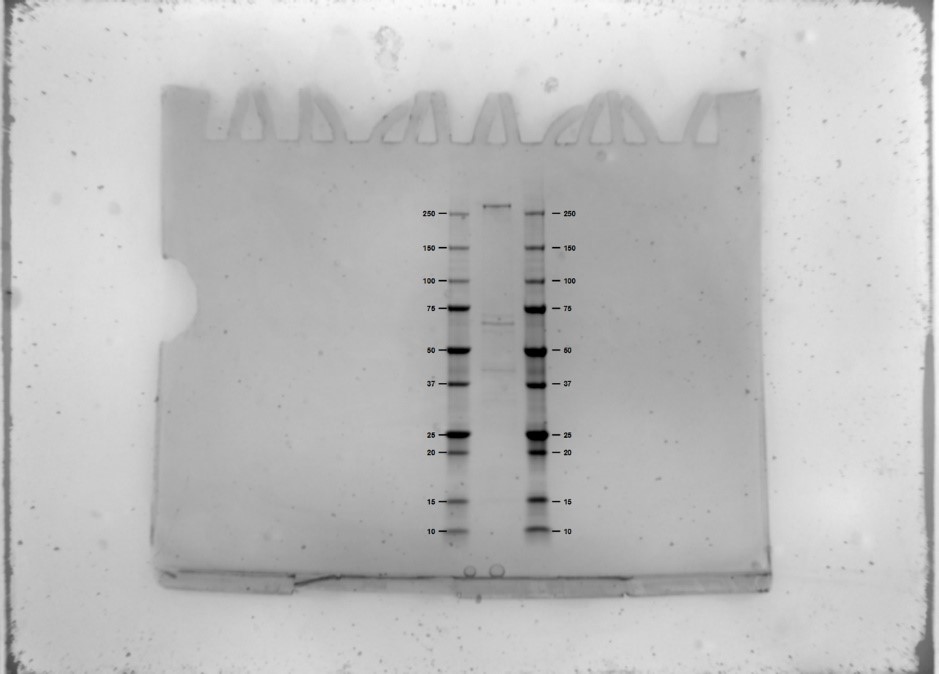

Supplement: Figure S1A.jpg [file KVIR_A_2506500_SM4856.jpg]

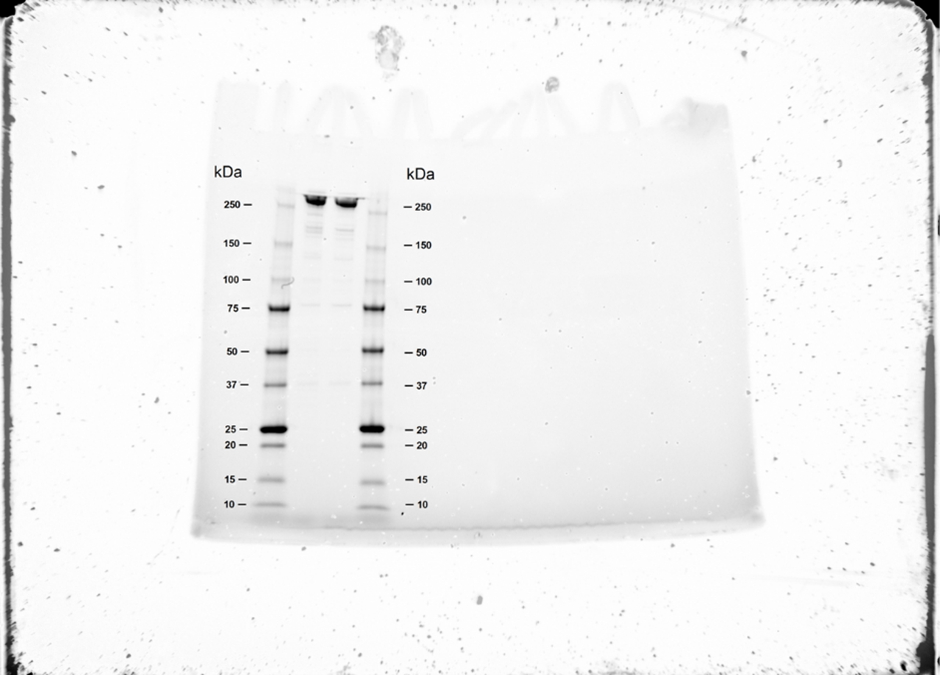

Supplement: Figure S1B.tif [file KVIR_A_2506500_SM4855.tif]
